# Phylogenetic relationship and comparative analysis of the main Bupleuri Radix species in China

**DOI:** 10.7717/peerj.15157

**Published:** 2023-04-14

**Authors:** Ping Wang, Jiqing Bai, Xue Li, Tiantian Liu, Yumeng Yan, Yichang Yang, Huaizhu Li

**Affiliations:** 1 Xianyang Normal University, Xianyang, China; 2 Shaanxi University of Chinese Medicine, Xianyang, China; 3 Xianyang Food and Drug Administration, Xianyang, China

**Keywords:** Bupleuri Radix, Genome skimming, *Bupleurum*, Plastome, Phylogeny

## Abstract

**Background:**

Bupleuri Radix (Chaihu) is a famous traditional Chinese medicine derived from *Bupleurum*, Apiaceae. The origin of cultivated Chaihu germplasm in China is unclear, which has led to unstable Chaihu quality. In this study, we reconstructed the phylogeny of the main Chaihu germplasm species in China and identified potential molecular markers to authenticate its origin.

**Methods:**

Three *Bupleurum* species (eight individuals), *B. bicaule*, *B. chinense*, and *B. scorzonerifolium*, were selected for genome skimming. Published genomes from *B. falcatum* and *B. marginatum* var. *stenophyllum* were used for comparative analysis.

**Results:**

Sequences of the complete plastid genomes were conserved with 113 identical genes ranging from 155,540 to 155,866 bp in length. Phylogenetic reconstruction based on complete plastid genomes resolved intrageneric relationships of the five *Bupleurum* species with high support. Conflicts between the plastid and nuclear phylogenies were observed, which were mainly ascribed to introgressive hybridization. Comparative analysis showed that noncoding regions of the plastomes had most of the variable sequences. Eight regions (*atp*F-*atp*H, *pet*N-*psb*M, *rps*16-*psb*K, *pet*A-*psb*J, *ndh*C-*trn*V/UAC and *ycf*1) had high divergence values in *Bupleurum* species and could be promising DNA barcodes for Chaihu authentication. A total of seven polymorphic cpSSRs and 438 polymorphic nSSRs were detected across the five Chaihu germplasms. Three photosynthesis-related genes were under positive selection, of which *acc*D reflected the adaptation fingerprint of *B. chinense* to different ecological habitats. Our study provides valuable genetic information for phylogenetic investigation, germplasm authentication, and molecular breeding of Chaihu species.

## Introduction

Bupleuri Radix (Chinese name: Chaihu), the dried root of *Bupleurum* species, has been used to treat cold, fevers, influenza, menstrual disorders, and hepatitis for more than 2,000 years (Yang et al., 2017; Zhu et al., 2017). Its primary use is as the main constituent of Chinese medicine prescriptions for soothing the liver and relieving depression, such as the Xiaochaihu Decoction, Chaihu Liver-soothing Powder, and Xiaoyao Pill (Yang et al., 2017; Zhu et al., 2017). *Bupleurum chinense* DC. and *B. scorzonerifolium* Willd. have been authenticated as the official botanical origin of Chaihu in the Chinese Pharmacopoeia (National Pharmacopoeia Committee, 2020), and are referred to as “Bei Chaihu” and “Nan Chaihu”, respectively. More than 20 *Bupleurum* species are habitually utilized as Chaihu due to their morphological similarity (Pan et al., 2002). As one of the most popular medicines in China, the demand for Chaihu in prescriptions and exports is enormous, while the supply of wild *Bupleurum* herb is limited. Artificial planting of Chaihu has become the dominant agriculture industry in some regions, such as the Chencang district in Shaanxi Province (Ma et al., 2020). However, the use of multiple *Bupleurum* species in different districts has also contributed to the complexity and diversity of cultivated Chaihu germplasms.

Based on plantation surveys, the cultivated germplasms of Chaihu in China are mainly from five species, *B. bicaule* Helm, *B. chinense*, *B. falcatum* L., *B. marginatum* var. *stenophyllum* (Wolff) Shan et Y. Li, and *B. scorzonerifolium* (Xu et al., 2014; Zhang et al., 2021; Zhang et al., 2022). Among these species, *B. chinense* is the most common species cultivated in China (Ma et al., 2020; Zhang et al., 2021). As a widespread species, *B. chinense* exhibits broad intraspecific morphological variation and different levels of medicinal quality under different growing conditions. The unclear origins of wild and cultivated Chaihu resources result in somewhat unstable Chaihu qualities in the Chinese market. Previous studies have greatly improved our understanding of *Bupleurum* based on morphology (Pan et al., 2002; Sheh & Watson, 2005), chromosome counts (Ma et al., 2015), nuclear ribosomal internal transcribed spacer (nrITS) (Chao et al., 2014; Neves & Watson, 2004; Xie et al., 2009), and plastid DNA markers (Wang et al., 2008; Wang, Ma & He, 2011). However, because of limited genetic information, relationships among some closely related species could not be fully resolved, such as *B. bicaule* and *B. scorzonerifolium*.

With the development of high-throughput sequencing technologies, genetic information from both nuclear and organellar DNA has provided an opportunity for addressing problems that have remained unresolved using traditional molecular systematics approaches (Liu et al., 2021a; Liu et al., 2021b). Chloroplast, the plant organelle for photosynthesis and carbon fixation, provides valuable genetic information for phylogenies of plants due to its low nucleotide substitution rates and uniparental inheritance (Davis, Xi & Mathews, 2014; Delsuc, Brinkmann & Philippe, 2005; Thode, Lohmann & Sanmartín, 2020). Compared to plastid barcodes, whole-plastome sequences could generate higher phylogenetic resolution and improve species identification (Fu et al., 2022; Meng et al., 2021; Wikström, Bremer & Rydin, 2020). Considering conflicts between chloroplast and nuclear phylogenies, complementary phylogenetic trees inferred with different datasets are necessary (Meng et al., 2021). Some regions of the nrDNA repeat, like ITS are widely used as phylogenetic markers for lower-level plant identification (Baldwin et al., 1995; Liu et al., 2021a; Liu et al., 2021b; Yao et al., 2010). Genome skimming, which relies on next generation sequencing, is a kind of shallow genome sequencing that could capture the plastome, nuclear repeat, and mitogenome regions (Dodsworth, 2015). Benefitting from abundant datasets and cost-efficiency, genome skimming has been used successfully in phylogenetic reconstruction at various levels, especially for taxonomically complex groups (Dodsworth, 2015; Straub et al., 2012).

In this study, we used the genome skimming approach to obtain the complete chloroplast genomes and nrDNA of *B. bicaule*, *B. chinense*, and *B. scorzonerifolium*, together with published genomes of two other *Bupleurum* species for comparative analysis. We aimed to: (1) explore the phylogenetic relationships of the five *Bupleurum* species that are the germplasm sources for Chaihu in China; and (2) select potential markers for authentication of Chaihu germplasm by comparative analysis. This study will provide valuable genetic information and potential markers for authentication of cultivated *Bupleurum* species, which will be helpful for cultivation and quality control of Chaihu.

## Materials & Methods

### Sample material

Three wild *Bupleurum* species, *B. bicaule* (one, BB01), *B. chinense* (five, BC01-05), and *B. scorzonerifolium* (two, BS01-02) were collected from different districts in China (Table 1)*.* Field experiments were approved by the Research Council of Xianyang Normal University (project number: 2019012). Fresh and healthy leaves were dried with silica gel, and then stored at −80 °C for DNA extraction. All species were authenticated by Director Xue Li (Xianyang Food and Drug Administration) and deposited at the Herbarium of Xianyang Normal University (http://www.xysfxy.cn/) accession Nos. XSYH202000101-XSYH202000801 (Table S1). Sampling information is shown in Table 1. To obtain the sequences of *B. falcatum* and *B. marginatum* var. *stenophyllum* for a broader comparison, we retrieved their raw sequenced reads from GenBank (*B. falcatum*, BF01: SRR12513791 and *B. marginatum* var. *stenophyllum*, BM01: SRR13195839), and used them for genome assembly and annotation as with the accessions sequenced in the study.

**Table 1:** Sampling information and summary of chloroplast genome features of *Bupleurum chinense*, *B. scorzonerifolium*, and *B. bicaule*.

Taxa	Code	Locality	Longitude, Latitude	Genome size (bp)	LSC length (bp)	SSC length (bp)	IR length (bp)	Unique genes	Unique CDS	tRNA	rRNA	Total GC content %	GC content of LSC (%)	GC content of SSC (%)	GC content of IR (%)	Herbarium accession
*B. chinense*	BC01	Heihe, Shaanxi	108.1754, 34.0188	155659	85551	17508	26300	113	79	30	4	37.7	35.8	31.4	42.8	XSYH2022000101
*B. chinense*	BC02	Danfeng,Shaanxi	110.5020, 33.9004	155579	85505	17496	26289	113	79	30	4	37.7	35.8	31.4	42.8	XSYH2022000201
*B. chinense*	BC03	Longde, Ningxia	106.2090, 35.4868	155612	85461	17499	26326	113	79	30	4	37.7	35.8	31.4	42.8	XSYH2022000301
*B. chinense*	BC04	Lanzhou, Gansu	103.6524, 36.4385	155540	85426	17492	26311	113	79	30	4	37.7	35.8	31.4	42.8	XSYH2022000401
*B. chinense*	BC05	Xizhou, Shanxi	112.4409, 38.6399	155573	85449	17510	26307	113	79	30	4	37.7	35.8	31.4	42.8	XSYH2022000501
*B. scorzonerifolium*	BS01	Ansai, Shaanxi	109.2021, 36.7978,	155828	85597	17597	26317	113	79	30	4	37.7	35.8	31.3	42.8	XSYH2022000601
*B. scorzonerifolium*	BS02	Fugu,Shaanxi	110.7683, 39.0646	155848	85612	17602	26317	113	79	30	4	37.7	35.8	31.3	42.8	XSYH2022000701
*B. bicaule*	BB01	Hulunbuir, Neimeng	118.1399, 47.6237	155866	85630	17602	26317	113	79	30	4	37.7	35.8	31.3	42.8	XSYH2022000801

### DNA extraction and sequencing

Total DNA was extracted using the CTAB method. Then, the extracted DNA (about 1 µg) was randomly sheared by Covaris (Brighton, UK), yielding fragments with an average size of 200–400 bp for library construction. Genome skimming sequencing was performed on the BGISEQ-500 platform with paired-end runs (BGI, Shenzhen, China).

### Genome assembly and annotation

High-throughput sequencing produced approximately 2 Gb raw data per sample. Raw reads were filtered and then assembled *de novo* into contigs using SOAPdenovo2 (Luo et al., 2012). To isolate plastid sequences, the generated contigs were pooled and mapped to the reference plastome (*B. chinense*: MN337347) using Bowtie2 (Langmead & Salzberg, 2012), then assembled to scaffolds by SPAdes v.3.11.1 (Bankevich et al., 2012). Geneious Prime (Kearse et al., 2012) was used to align the scaffolds with the aforementioned reference, resulting in the final plastid genomes. Annotation of the assembled sequences was also performed in Geneious Prime with *B. chinense* (MN337347) as a reference. All plastomes were deposited in GenBank with the accession numbers shown in Table S1.

### Comparative analysis and sequence divergence analysis

For comparative analysis, MAFFT v.7 (Katoh et al., 2002) was used to align the plastomes of *Bupleurum* individuals. The nucleotide substitution numbers and sequence identities of *Bupleurum* plastomes were calculated with Geneious Prime. The IR expansion/contraction of five *Bupleurum* species was tested by comparing the IR borders and neighboring genes of ten chloroplast genomes using an IRscope online program (Amiryousefi, Hyvönen & Poczai, 2018). To detect and characterize the divergence hotspots, mVISTA (Frazer et al., 2004) was used to compare the complete plastid genomes of five *Bupleurum* species with *B. chinense* (BC01) as a reference. The nucleotide diversity (Pi) among the plastid genome sequences was calculated using sliding window analysis in DnaSP v.5 (Librado & Rozas, 2009). The window size was 600 bp, and the step size was 200 bp. The single nucleotide polymorphisms (SNPs) and insertions-deletions (InDels) were identified using MEGA v.6 (Kumar et al., 2018) and visualized using Circos 0.64 (Krzywinski et al., 2009).

Simple sequence repeats in the chloroplast genome sequences (cpSSRs) were identified *via* Perl script in MISA (Thiel et al., 2003) with respective thresholds of 10, 5, 4, 3, 3, 3 for mono-, di-, tri-, tetra-, penta-, and hexanucleotides. The candidate polymorphic cpSSRs and nuclear SSRs (nSSRs) were identified using CandiSSR (Xia et al., 2016). The nuclear contigs were used to generate nSSRs by removing the plastid and mitochondrial contigs from the original contigs using Bowtie2 with *B. chinense* as a reference (GenBank accession numbers MN337347 and OK166971 for chloroplast and mitochondrial genomes, respectively). The parameters implemented in CandiSSR were as follows: flanking sequence length of 100, blast e-value cutoff of 1e^−10^, BLAST identity cutoff of 95, and BLAST coverage cutoff of 95.

### Gene selective pressure analysis

To detect whether plastid genes were under selection pressure, the ratio of the non-synonymous (*Ka*) to the synonymous substitution rate (*Ks*) was calculated for all protein-coding genes by DnaSP. Genes were considered to undergo positive selection when the value of *Ka*/*Ks* was higher than 1, while *Ka*/*Ks* < 1 indicated that the genes were under neutral selection (Yang & Nielsen, 2002).

### Phylogenetic analysis

For phylogenetic inference, maximum likelihood (ML) and Bayesian inference (BI) analyses were performed for three different datasets: (1) complete plastome sequences, (2) all shared protein-coding sequences (CDS), and (3) nrDNA sequence data (ITS). For phylogenetic analysis based on plastid data, we selected 32 plastid genomes, including eight accessions of three species we sequenced, two accessions (MT797174 and MT075712) we reassembled and reannotated, 20 *Bupleurum* accessions retrieved from NCBI, together with two accessions as outgroups (*Chamaesium paradoxum* MK780227, *Pleurospermum rivulorum* MW147504).

To obtain nrDNA data, the original contigs of the accessions studied in the plastid phylogeny were mapped to the ITS sequence of *B. chinense* (MH710800) and extracted by Bowtie2. The species used in the plastid and nrDNA datasets were kept largely consistent. For species without nrDNA data, the ITS sequences were downloaded according to Wang, Ma & He (2011). Finally, 49 nrITS sequences of *Bupleurum* accessions, together with *Pleurospermum hookeri* var. *thomsonii* HQ824799 and *P. rivulorum* HQ824798, were selected for the phylogenetic reconstruction.

The software jModelTest v.2.1.4 (Posada, 2008) was used to determine the best-fit nucleotide substitution model, resulting in GTR+I+G for the plastid datasets and GTR+G for the nrITS data under the Akaike Information Criterion (AIC). ML analyses were conducted using RAxML v.8.2.11 (Stamatakis, 2014) with 1,000 bootstrap iterations. BI analyses were performed in MrBayes v.3.2.6 (Ronquist & Huelsenbeck, 2003). Four Markov chains starting with a random tree were run simultaneously for 5,000,000 generations, followed by a sampling point at every 1,000 generations. The resulting trees were visualized using FigTree v.1.4.4 (Rambaut, 2018). Bootstrap support (BS) value and posterior probability (PP) were used to evaluate the feasibility of each branch for ML and BI, respectively.

## Results

### Chloroplast genome structure and characteristics analyses

**Figure 1:**
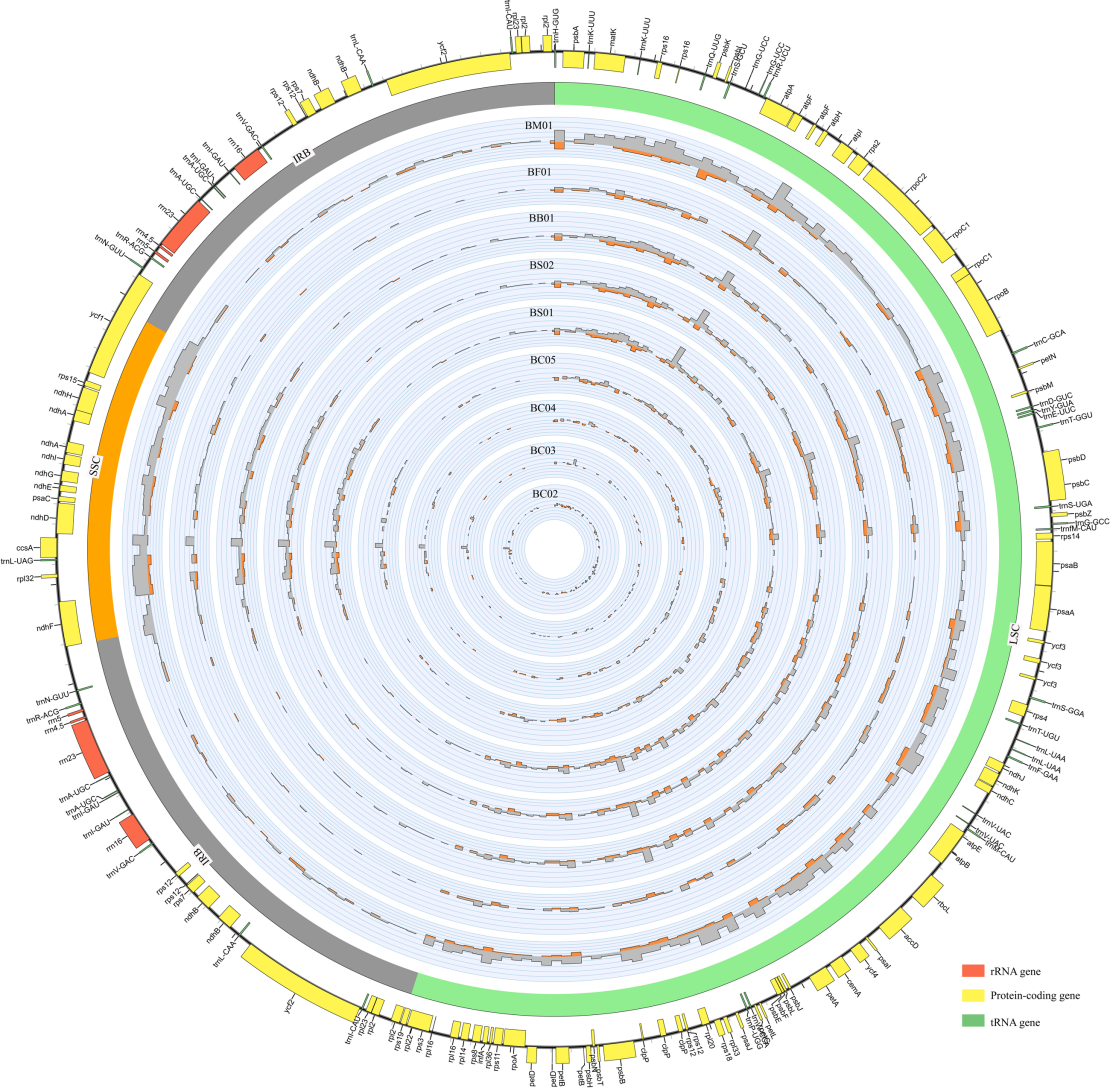
An overview of gene distribution and genome variation among *Bupleurum* plastomes using Circos. Genes located outside and inside of the outer layer circle represent clockwise and counterclockwise transcription, respectively. The inner circles exhibit the SNPs and InDels every 600 bp across five *Bupleurum* species with *B. chinense* (BC01) as a reference. The histograms of SNPs are outside the circles, while the histograms of InDels are inside the circles.

Plastid sequences were successfully assembled for ten accessions, of which *B. falcatum* (MT797174, BF01) and *B. marginatum* var. *stenophyllum* (MT075712, BM01) were reassembled. For *Bupleurum* species sequenced in this study, the complete plastomes ranged from 155,540 bp in BC04 to 155,866 bp in BB01. Sequences of the *B. chinense* plastomes ranged from 155,540 bp to 155,659 bp, while those from *B. scorzonerifolium* ranged from 155,828 bp to 155,848 bp. All chloroplast genomes exhibited a typical quadripartite structure, consisting of a large single-copy (LSC) region (85,426–85,630 bp) and a small single-copy (SSC) region (17,492–17,602 bp) separated by two inverted regions (IRs, each with 26,289–26,326 bp) (Table 1, Fig. 1). The overall GC content of each plastome was 37.7%, whereas the GC contents in the LSC, SSC, and IR were 35.8%, 31.3–31.4%, and 42.8%, respectively.

Each *Bupleurum* chloroplast genome encoded a total of 132 genes, of which 113 were unique, containing 79 protein-coding genes (CDS), 30 transfer RNA genes (tRNA), and four ribosomal RNA genes (rRNA). The different functional genes were divided into three categories, namely self-replication, photosynthesis, and other (Table S2). A total of 19 genes were duplicated in the IRs, including six CDSs (*rps* 12, *rps* 7, *ndh* B, *ycf* 2, *rpl* 23, and *rpl* 2), seven tRNAs (*trn* N-GUU, *trn* R-ACG, *trn* A-UGC, *trn* l-GAU, *trn* V-GAC, *trn* L-CAA, and *trn* l-CAU), four rRNAs (*rrn* 4.5, *rrn* 5, *rrn* 23, and *rrn* 16), and two pseudogenes (*ycf* 1 and *rps* 19) with incomplete copies. The *rps* 12 gene was a particular trans-splicing gene with the 5′-end exon located in the LSC region, while two copies of the 3′-end exon were situated in the IR. There were 18 genes containing introns, including six tRNAs and 12 CDSs. Among these, *ycf* 3, *clp* P, and *rps* 12 had two introns. The gene *trn* K-UUU had the largest intron with 2,531 bp, where the *mat* K gene was located.

### Comparisons of the *Bupleurum* plastomes

Differences and evolutionary divergences were tested based on ten *Bupleurum* plastomes, including five *B. chinense*, two *B. scorzonerifolium*, one *B. bicaule*, one *B. falcatum*, and one *B. marginatum* var. *stenophyllum*. The ten *Bupleurum* plastomes exhibited a high level of sequence similarity and structure (Table 2). The percentage of identity was 96.72–99.97%, with 49-5,160 nucleotide differences. Among *B. chinense* plastomes, the identity narrowly ranged from 99.4% to 99.81%. At the interspecies level, *B. chinense* (BC01) and *B. marginatum* var. *stenophyllum* (BM01) exhibited the most significant sequence difference with 96.72% identity (Table 2).

**Table 2:** Numbers of nucleotide substitutions (upper) and sequence identity (lower) in ten plastomes of five *Bupleurum* species.

	BC01	BC02	BC03	BC04	BC05	BS01	BS02	BB01	BF01	BM01
BC01		451	293	522	930	2131	2155	2165	1499	5160
BC02	99.71		415	438	869	2082	2114	2126	1441	5121
BC03	99.81	99.73		456	869	2062	2098	2106	1476	5141
BC04	99.67	99.72	99.71		924	2126	2159	2167	1436	5142
BC05	99.4	99.44	99.44	99.41		2034	2064	2078	1517	5139
BS01	98.64	98.67	98.68	98.64	98.7		224	243	1733	5062
BS02	98.62	98.65	98.66	98.62	98.68	99.86		49	1731	5050
BB01	98.62	98.64	98.65	98.62	98.67	99.84	99.97		1745	5065
BF01	99.04	99.08	99.06	99.08	99.03	98.89	98.89	98.89		4947
BM01	96.72	96.74	96.73	96.73	96.73	96.78	96.79	96.78	96.85	

**Figure 2:**
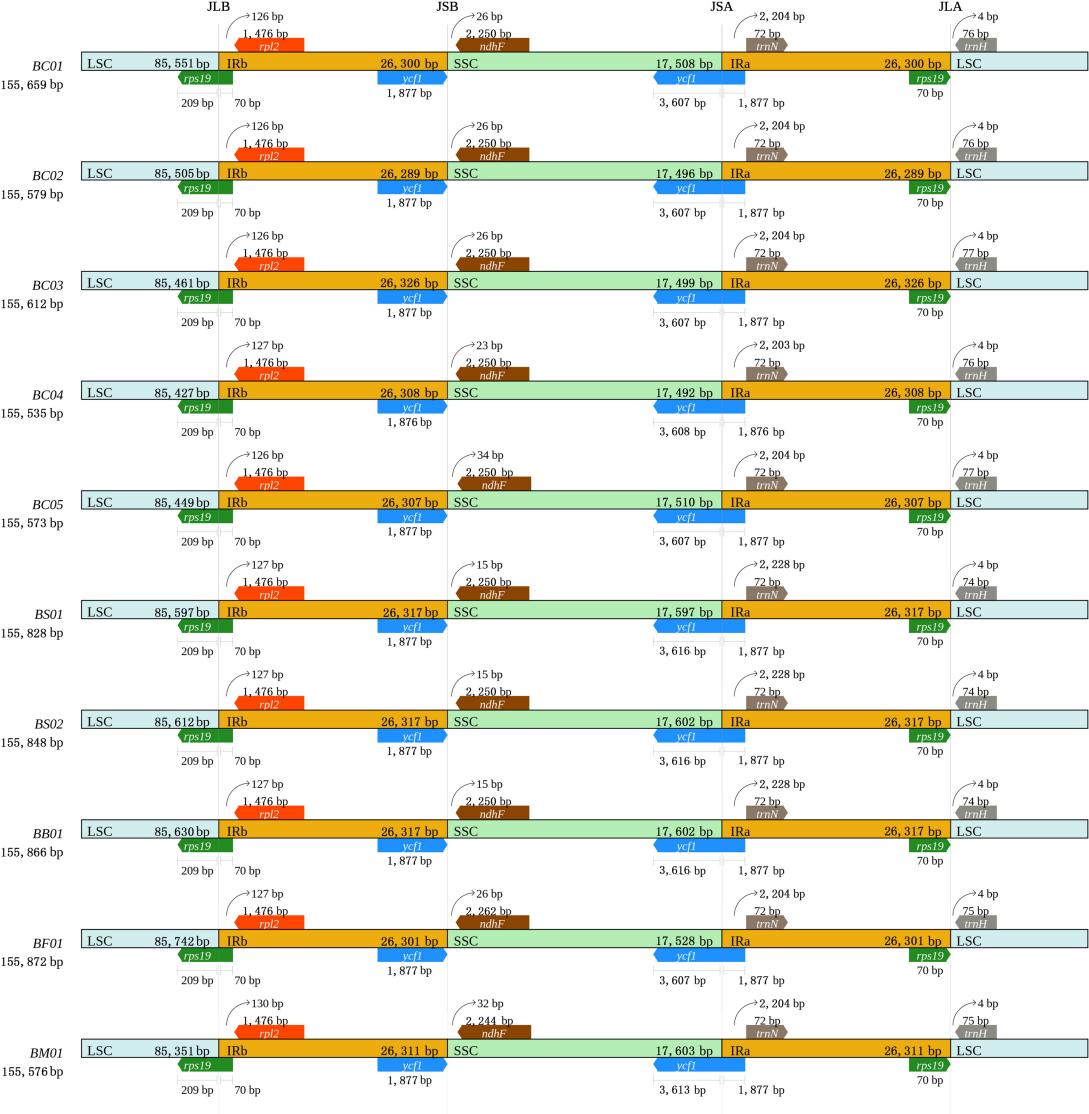
Comparison of the IR border regions among the ten plastid genomes of five *Bupleurum* species.

To further observe the contraction and expansion of IR, the exact IR border positions and their adjacent genes among the *Bupleurum* plastomes were compared (Fig. 2). Genes *rps* 19, *rpl* 2, and *trn* H were in the junctions of LSC/IR, while *ycf* 1, *ndh* F, and *trn* N flanked the junctions of SSC/IR. Genes in the junction regions were the same length, except the *ycf* 1 gene, which ranged from 5,483 bp to 5,490 bp. In all ten *Bupleurum* plastomes, *rps* 19 crossed the LSC/IRb region with 279 bp in length, of which 70 bp occurred in the IRb region. The pseudogene fragment of *rps* 19 was located aside the IRa/LSC junction with 70 bp. The *rpl* 2 gene was separated from the LSC/IRb junction with 126–127 bp in *B. chinense*; 127 bp in *B. bicaule*, *B. falcatum*, and *B. scorzonerifolium*; and 130 bp in *B. marginatum* var. *stenophyllum.* The *trn* H gene was 4 bp away from the IRa/LSC region in all ten *Bupleurum* plastomes. In the SSC/IR junctions, *ycf* 1 crossed the SSC/IRa junction, and the pseudogene fragment narrowly ranged from 1,876–1,877 bp in IRb. The length of the *ycf* 1 gene was 5,484 bp in *B. chinense* and *B. falcatum,* while it was 5,483 bp in *B. bicaule and B. scorzonerifolium*, and 5,490 bp in *B. marginatum* var. *stenophyllum*. The *ndh* F gene was 26–34 bp away from the IRb/SSC junction, and the *trn* N gene was 2,204–2,228 bp away from the SSC/IRa junction.

### Sequence variation analysis

**Figure 3:**
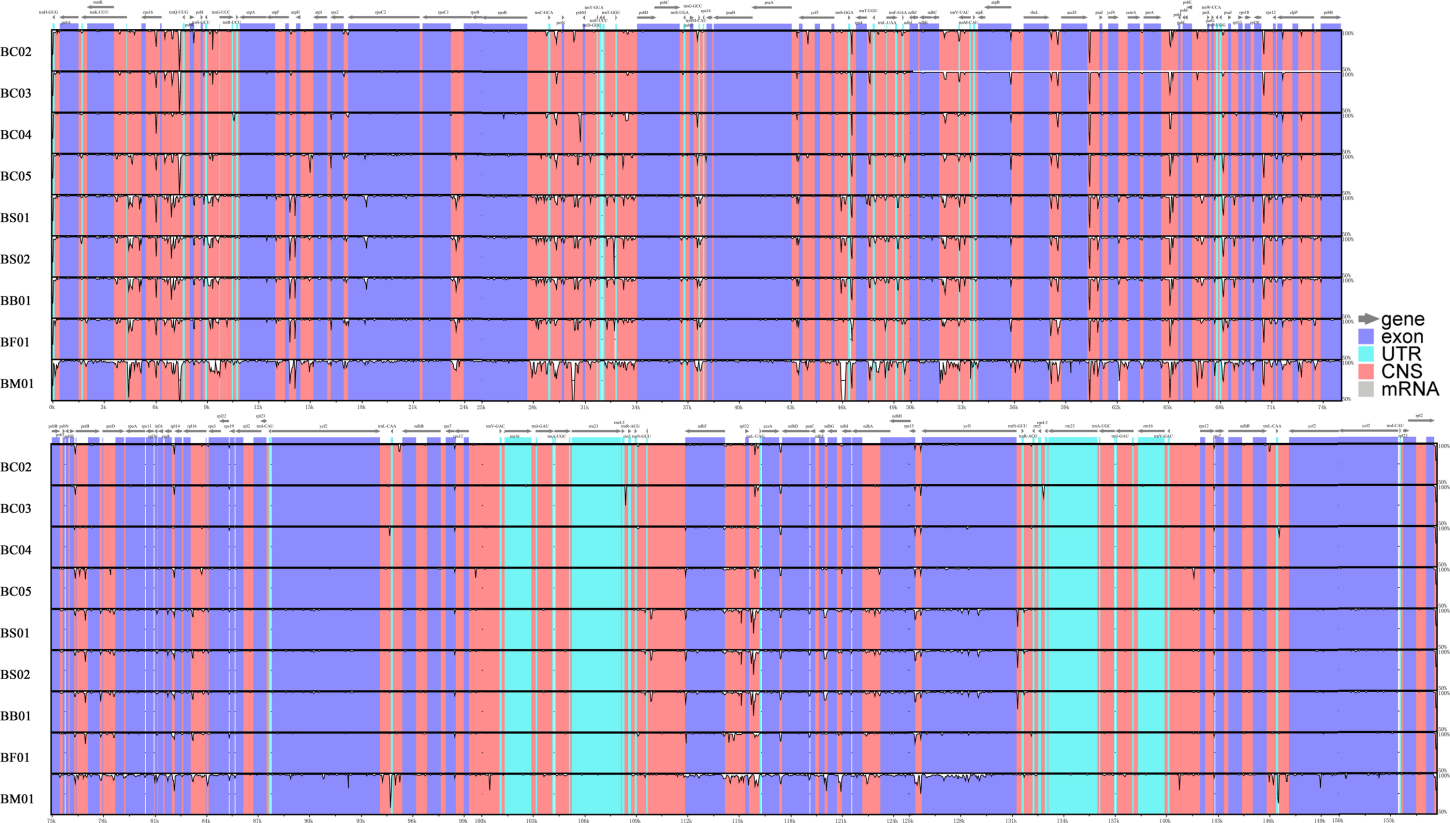
MVISTA-based sequence identity among the plastid genomes of five *Bupleurum* species with *B. chinense* (BC01) as a reference.

**Figure 4:**
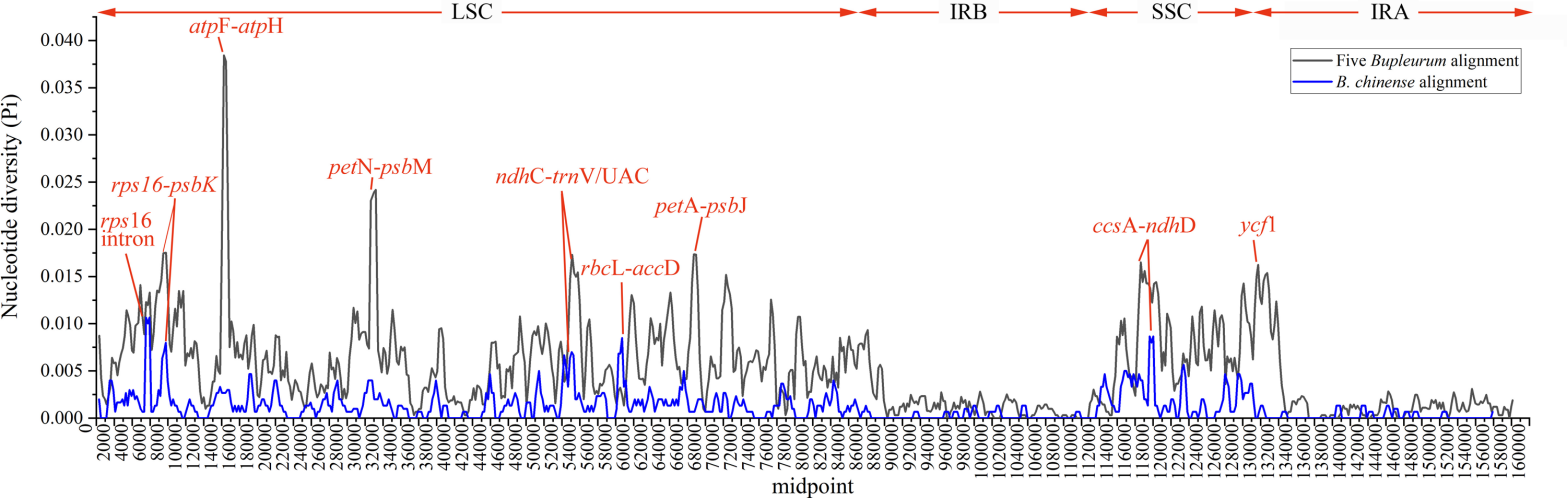
Nucleotide diversity (Pi) among the plastomes with sliding window analysis (window length: 600 bp). The *y*-axis represents nucleotide diversity of the alignment of five *Bupleurum* species (in black) and the *B. chinense* alignment (in blue), while the *x*-axis represents the position of the window midpoint.

Abundant polymorphic sites were observed among the five *Bupleurum* species. In addition, *B. chinense* sequences also exhibited intraspecies divergence. With *B. chinense* (BC01) as a reference, the mVISTA result indicated that noncoding sequences exhibited a higher level of divergence than CDS, and most of the sequences were situated in the LSC and SSC regions (Fig. 3). We characterized genomic polymorphisms of the *Bupleurum* plastomes, including SNPs and InDel-variable loci (Fig. 1, Table S3). At the interspecies level, the number of SNPs varied from 420 (BF01 *vs.* BC01) to 2,091 (BM01 *vs.* BC01), and the InDel loci varied from 163 (BF01 *vs.* BC01) to 340 (BM01 *vs.* BC01). At the intraspecies level, the numbers of SNPs and InDels among *B. chinense* plastomes ranged from 70–303 and 39–114, respectively. The LSC regions harbored the largest number of genomic polymorphism sites, followed by SSC regions (Fig. 4). The IRs were conserved among *Bupleurum* species. The nucleotide diversity (Pi) of the chloroplast genomes was calculated to assess the sequence divergence level. Among all ten *Bupleurum* species, *atp* F-*atp* H, *pet* N-*psb* M, *rps* 16-*psb* K, *pet* A-*psb* J, *ndh* C-*trn* V/UAC, and *ycf* 1 had relatively higher divergence values (Pi >0.015). Chloroplast genome diversity at the intraspecies level of *B. chinense* was also detected, of which *rps* 16 intron, *ccs* A-*ndh* D, *rbc* L-*acc* D, *rps* 16-*psb* K, and *ndh* C-*trn* V/UAC had high Pi values.

**Figure 5:**
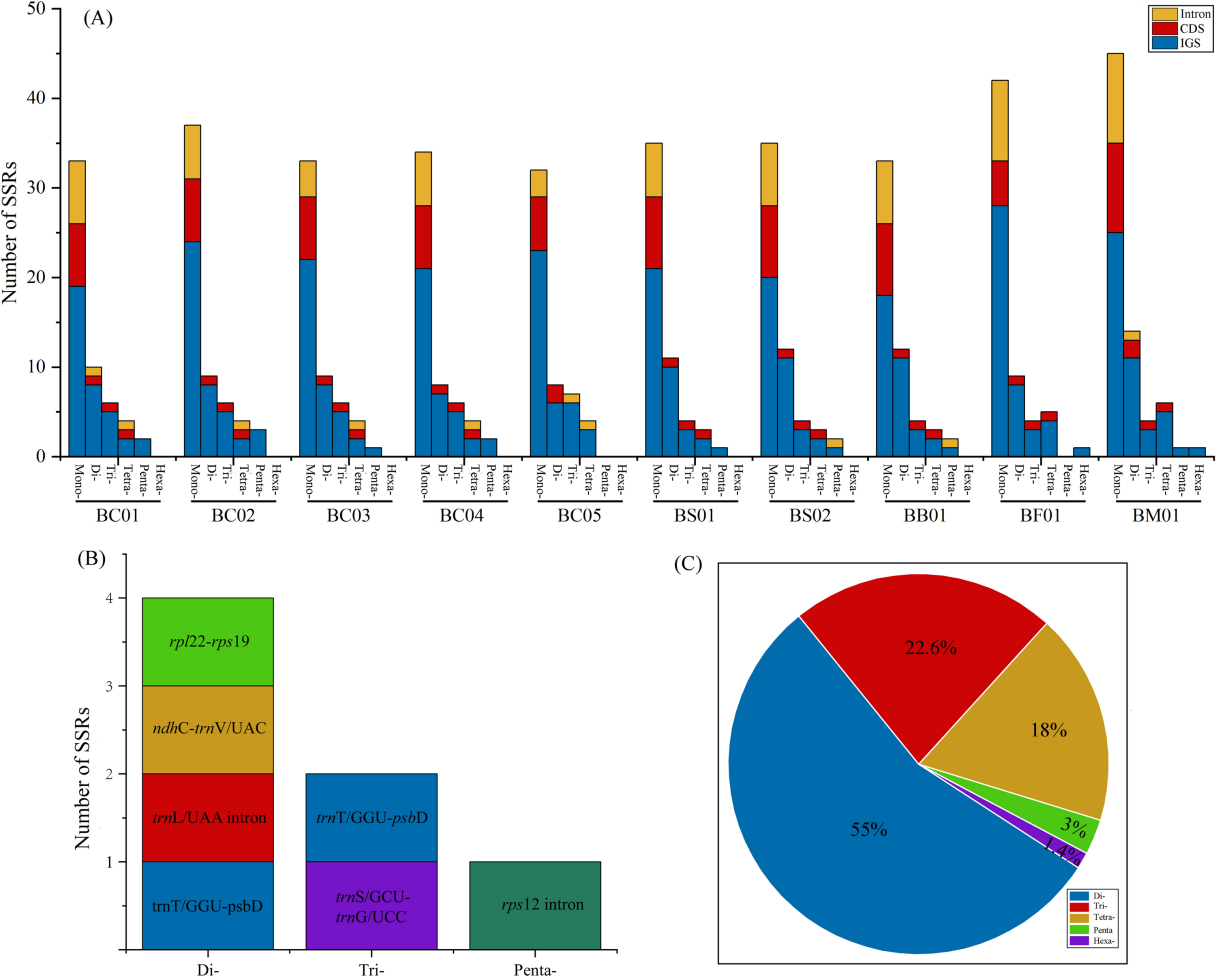
Analysis of SSRs in the five *Bupleurum* species. (A) Number and location of six types of cpSSRs in ten plastomes of the five *Bupleurum* species. (B) Seven polymorphic cpSSRs across five *Bupleurum* species identified using CandiSSR. (C) Polymorphic nSSRs across five *Bupleurum* species identified using CandiSSR.

Simple sequence repeats (SSRs) analysis showed that the total number of cpSSR loci in plastomes ranged from 57 (BC05) to 81 (BM01) (Fig. 5A, Table S4). As shown in Fig. 5A, the SSRs were mainly located in the intergenic spacer (IGS), followed by CDS and intron regions. Monomers were the primary type, accounting for 60–68.9% of the SSRs in ten *Bupleurum* plastomes. As the patterns of SSR distribution were similar among the ten *Bupleurum* plastomes, BC01 was chosen as an example to exhibit the SSR characters. A total of 60 SSRs were detected in BC01, of which 58.3% were monomers, 18.3% were dimers, 13.4% were trimers, 6.7% were tetramers, 3.3% were pentamers, and no hexamers were present. Hexamers were only found in BF01 and BM01. Moreover, 65% of the SSRs were located in the IGS region, followed by the CDS region (20%) and the intron (15%). The genes *rps* 16-*psb* K, *rps* 2-*rpo* C2, *trn* T/GGU-*psb* D, and *ycf* 1 harbored more than two SSRs in each *Bupleurum* plastome. By comparing the cpSSRs across the five *Bupleurum* species using CandiSSR, a total of seven polymorphic cpSSRs were identified (Fig. 5B, Table S5). Specifically, four were dimers, two were trimers, and one was a pentamer. All polymorphic cpSSRs were detected in noncoding regions, of which *trn* T/GGU-*psb* D harbored two polymorphic cpSSRs. Additionally, 438 candidate polymorphic nSSRs were identified from the nuclear contigs of the five *Bupleurum* species (Fig. 5C, Table S6). More specifically, dimers were the primary type of polymorphic nSSR (55%), followed by trimers (22.6%) and tetramers (18%). Polymorphic pentamers and hexamers accounted for 3% and 1.4%, respectively.

### Selective pressure analysis

**Figure 6:**
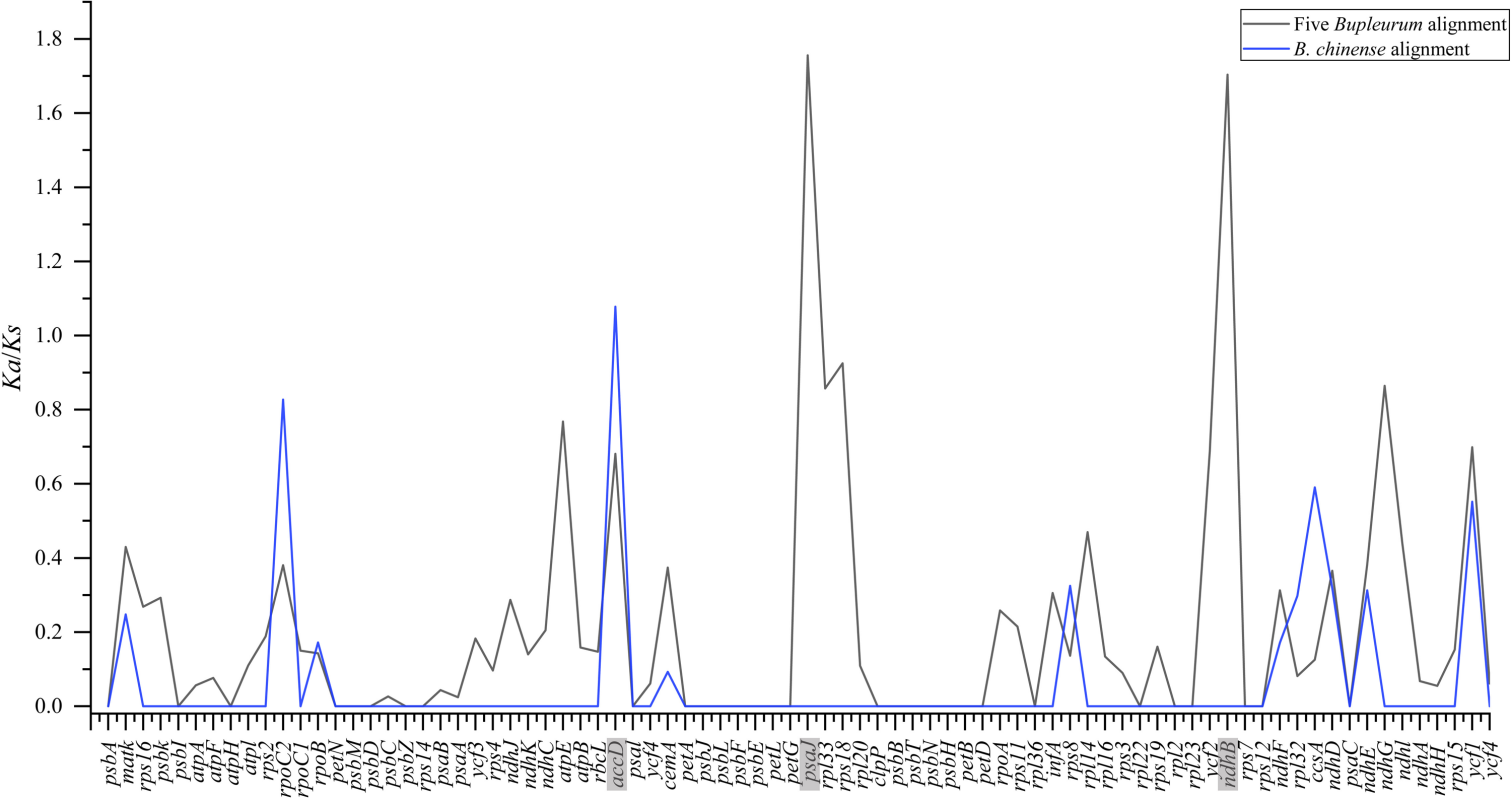
*Ka*/*Ks* values of 79 shared coding genes in the alignment of five *Bupleurum* species (in black) and the *B. chinense* alignment (in blue). The genes with *Ka*/*Ks* values larger than one are in grey.

To pinpoint whether genes underwent adaptive evolution in *Bupleurum* plastomes, the *Ka*/*Ks* values of the protein-coding genes were calculated. At the interspecies level, the *Ka*/*Ks* values of *psa* J and *ndh* B were greater than 1, indicating that the corresponding genes experienced positive selection (Fig. 6). The *Ka*/*Ks* values of the photosynthetic genes and self-replication genes were 0.1853 ± 0.3804 and 0.1926 ± 0.2377, respectively. Both were lower than those of other genes (0.4127 ± 0.2489). Among the *B. chinense* plastomes, *acc* D related to the subunit of acetyl-CoA-carboxylase had the highest of *Ka*/*Ks* (1.076), followed by *rpo* C2 (*Ka*/*Ks* = 0.827) which is related to self-replication (Fig. 6).

### Phylogenetic analysis

**Figure 7:**
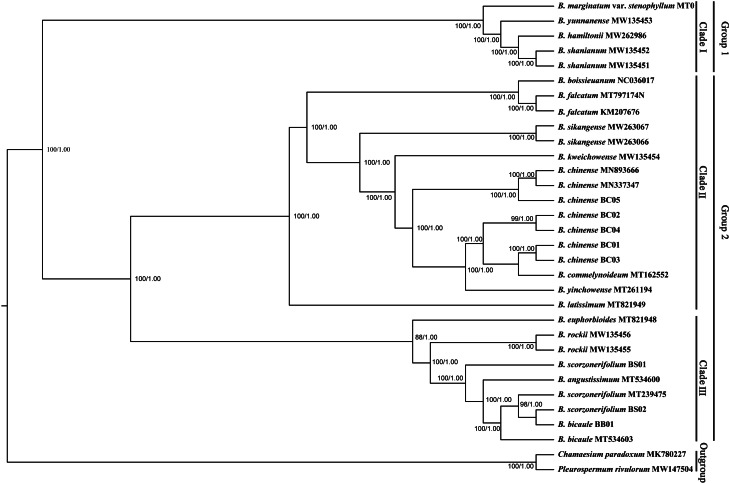
Phylogenetic tree reconstructed using maximum likelihood (ML) and Bayesian inference (BI) based on 32 complete plastid genomes. Numbers shown at the corresponding nodes represent ML bootstrap support (BS) values and Bayesian posterior probabilities (PP), respectively.

Phylogenetic trees based on complete plastid genomes and the CDS showed the same topology, therefore, only the tree based on the complete plastid genomes with a slightly higher bootstrap support value was shown. All *Bupleurum* accessions were clustered into two main groups with absolute support (Fig. 7). *B. marginatum* var. *stenophyllum* (MT075712) and the other three congeneric species that are mainly distributed in Southwestern China formed a monophyletic group, appearing as an early branching clade within *Bupleurum* (Clade I). The remaining accessions could be divided into two subgroups, Clade II and Clade III (Fig. 7). *B. chinense* accessions and the *B. falcatum* accession were both in Clade II. *B. chinense* grouped with *B. commelynoideum* and *B. yinchowense* forming a clade with absolute support. Within Clade III, morphologically similar species *B. angustissimum*, *B. bicaule*, and *B. scorzonerifolium* were clustered together, sister to *B. rockii* and *B. euphorbioides*.

**Figure 8:**
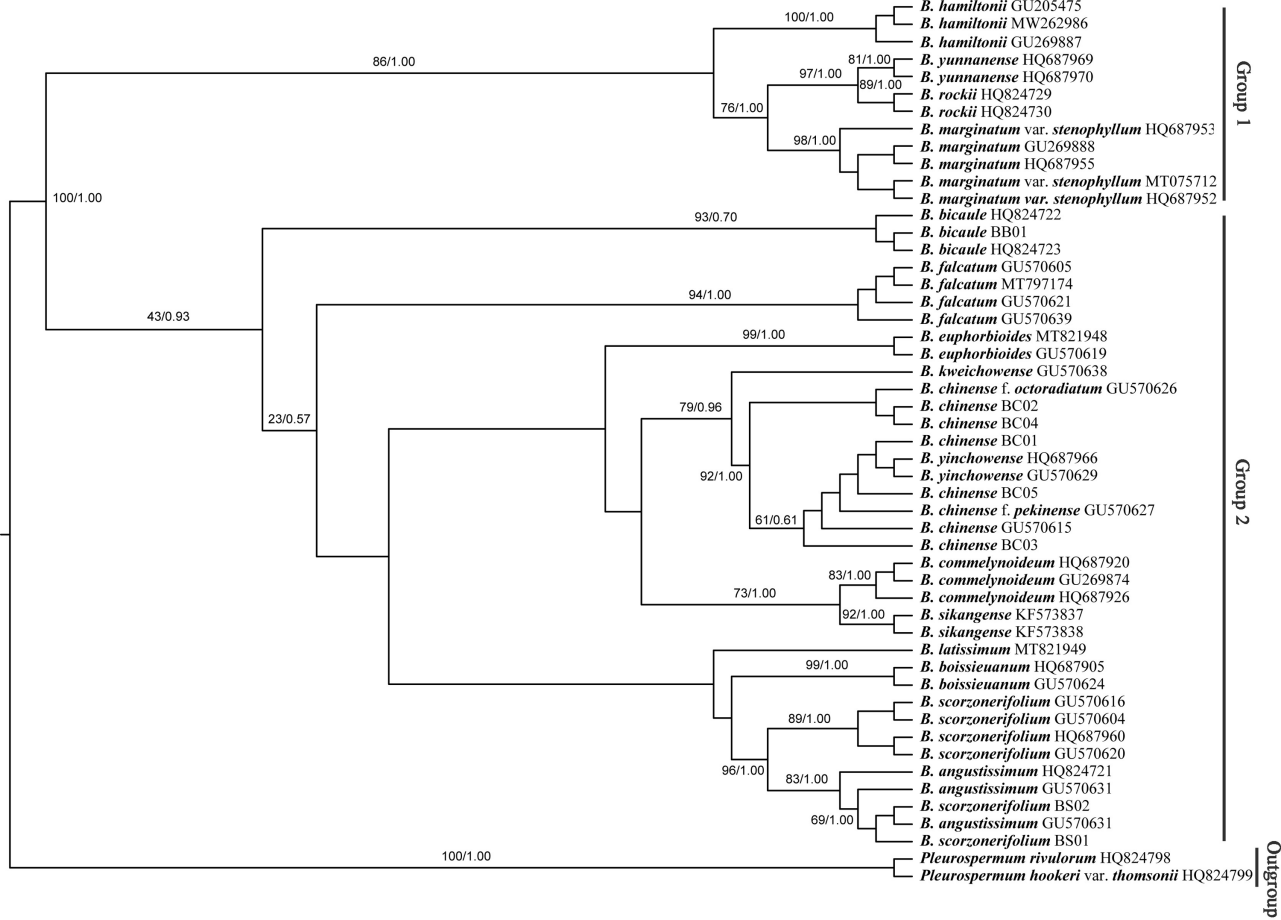
Phylogenetic tree reconstructed using maximum likelihood (ML) and Bayesian inference (BI) based on ITS. Numbers shown at the corresponding nodes represent BS/PP.

The *Bupleurum* accessions were also clustered into two groups based on ITS data (Fig. 8). Though some of the branch nodes were poorly supported, each *Bupleurum* species studied formed a monophyletic lineage with high support, except for *B. chinense, B. angustissimum*, and *B. scorzonerifolium*. However, incongruences were observed between the phylogenies based on nuclear ITS and chloroplast datasets. Based on ITS data, *B. chinense* and its forms were nested with *B. yinchowense*, while distinct from *B. falcatum.* The *B. scorzonerifolium* accessions from northwestern China were grouped with *B. angustissimum* as shown in the tree based on the chloroplast dataset. *B. bicaule* represented an independent lineage in the nuclear dataset.

## Discussion

### Infrageneric relationships within *Bupleurum* and taxonomic implications

*Bupleurum* L. is one of the largest genera in Apiaceae, represented by 150–180 species, of which nearly 50 species occur in China (Sheh & Watson, 2005). Due to the similar morphological characteristics and broad intraspecific morphological variation under different ecological habitats, species delimitation based on traditional classification systems is extremely difficult in this genus. The complicated infrageneric relationships also result in problems with cultivation. Based on more extended sampling, Wang, Ma & He (2011) proposed to divide the Chinese *Bupleurum* into two groups. Specifically, one group contained species from the southwest, while another consisted of the species mainly distributed in northern China. However, the statistical support of the main clade was relatively low based on the combined dataset of *mat* K and *trn* H-*psb* A. In this study, the phylogenetic topology based on complete chloroplast genomes was congruent to the framework of Wang, Ma & He (2011), but with much higher internal resolution. Among the five Chaihu germplasm resources, *B. marginatum* var. *stenophyllum* clustered in the early branching group of the phylogenetic trees, separated from the other four *Bupleurum* species. Due to the high yield and high content of saikosaponins, *B. marginatum* var. *stenophyllum* was introduced from Tibet to Gansu and Shanxi Provinces in China with widespread planting (Liu et al., 2021a; Liu et al., 2021b). Modern pharmacological studies have shown that saikosaponins are not only the main bioactive components of Chaihu, but also the main toxic components (Huang & Sun, 2010; Lv et al., 2009; Zhou et al., 2021). Wang et al. (2020) demonstrated that *B. marginatum* var. *stenophyllum* contained higher content of saikosaponins A and D, and had a higher level of acute toxicity. Xia et al. (2021) compared the overall chemical components of *B. marginatum* var. *stenophyllum* and *B. chinense*, and showed clear species-clustering. Thus, the use of *B. marginatum* var. *stenophyllum* as Chaihu should be done with caution.

In the plastid and ITS trees, *B. chinense* accessions were clearly distinct from those of *B. scorzonerifolium*. Morphologically, the two species are distinguished by a combination of characteristics, including leaf veins, fibrous remnant sheaths, and color of the root bark. Notably, phylogenetic trees based on complete plastid genomes and nuclear ITS datasets both supported close affinities of *B. yinchowens* and *B. chinense*, and *B. angustissimum* and *B. scorzonerifolium*. The delimitation confusion has long been an issue in these species (Wang, Ma & He, 2011). Based on similar morphology and overlapping ecological distributions (Sheh & Watson, 2005), we proposed *B. yinchowens* as conspecific to *B. chinense*, and *B. angustissimum* as conspecific to *B. scorzonerifolium*, but more evidence was needed. *B. falcatum* showed a closer phylogenetic relationship with *B. chinense* in the plastid trees, while the species was resolved as a distinct clade not closely related to *B. chinense* and *B. scorzonerifolium* but to *B. bicaule*. *B. falcatum* is a cultivated species originating from Japan, where it is the official botanical origin of Bupleuri Radix in the The Society of Japanese Pharmacopoeia (2011). Consequently, we speculated that introgressive hybridization during years of cultivation might contribute to the conflicts between plastid and nuclear ITS phylogenies. Theoretically, evolutionary processes including incomplete lineage sorting, introgressive hybridization, and paralogy conflation might contribute to phylogenetic incongruence among different datasets (Liu et al., 2020; Wendel & Doyle, 1998). Many studies suggested that introgressive hybridization was a prominent factor for phylogenetic incongruence at lower taxonomic levels. Based on the plastid datasets, *B. bicaule* and *B. scorzonerifolium* were clustered with *B. angustissimum*. Morphologically, these three species are highly similar, with fibrous remnant sheaths around the stem. However, *B. bicaule* accessions did not cluster with *B. angustissimum* and *B. scorzonerifolium* in the nrDNA tree. Considering the corresponding node had low support, we cannot exclude the possibility that insufficient information from ITS sequences resulted in the incongruence. Moreover, *B. bicaule* was considered to be involved in hybridization events to coexist with other *Bupleurum* species. The delimitation of *B. bicaule* needs further study.

### Conserved plastid genome structure

For the five *Bupleurum* species examined here, the chloroplast genomes displayed a typical quadripartite structure, as in most angiosperms (Palmer, 1985). The chloroplast genome structures were found to be largely conserved with lengths varying from 155,540 bp to 155,866 bp. Previous studies have reported that gene loss (Wakasugi et al., 1994; Wolfe, Morden & Palmer, 1992), expansion and contraction of the IRs (Lin et al., 2003; Parks, Cronn & Liston, 2009), and intergenic region variation (Tang et al., 2004; Wu et al., 2011) are three important factors driving size variation in the plastid genome. Across the five *Bupleurum* species in this study, the genes distributed around the junction boundaries slightly varied in length. Among these, the SSC/IR borders showed a higher level of differences than LSC/IR, in contrast to the study of Huang et al. (2021) in a broader comparison of *Bupleurum* species. As expected for the intraspecific divergence inferred from plastid genomes, the junction boundaries in *B. chinense* accessions were more convergent. We proposed that size variations in the chloroplast genome within *Bupleurum* were due to variation of intergenic regions.

### Variable regions for potential molecular markers

In this study, abundant sequences with SNPs and SSRs were detected across the five *Bupleurum* species. As expected, large-scale sequencing provided abundant variable sequences, which could be used for species authentication and phylogenetic inference. Our results showed that the majority of variable sequences were located in noncoding regions at either the interspecific level or the intraspecific level. Previous studies have demonstrated that noncoding sequences of the chloroplast genome perform well as phylogenetic markers, and in phylogenetic construction and population genetic studies (Shaw et al., 2007; Borsch & Quandt, 2009). In addition, the majority of noncoding sequences with high nucleotide diversity were situated in single-copy regions. Perry & Wolfe (2002) showed that the nucleotide mutation rate of single-copy regions was 2.3 times higher than that of the inverted repeats.

In this study, *atp* F-*atp* H, *pet* N-*psb* M, *rps* 16-*psb* K, *pet* A-*psb* J *ndh* C-*trn* V/UAC, and *ycf* 1 regions had high values of nucleotide diversity across the five *Bupleurum* species*. Atp* F-*atp* H has been proposed as a potential DNA barcode for identifying plant taxa at the species level (Lahaye et al., 2008; Thakur et al., 2019). The high nucleotide diversity of the remaining five genes has been reported in *Bupleurum* before (Huang et al., 2021; Li et al., 2020; Xie et al., 2021). In particular, *ycf* 1 has been proposed as the most promising plastid DNA barcode of land plants (Dong et al., 2015). The Pi values of the corresponding genes were slightly lower than that in Huang et al. (2021), mainly because of the limited *Bupleurum* species involved in our study.

Repeat units, distributed in genomes with high frequency, are essential in the rearrangement and divergence of the genome (Weng et al., 2014). Microsatellites (SSRs), containing repetitive sequences of 1–6 bp in length, are used as molecular markers for population genetics, phylogeography, and systematics (George et al., 2015). Owing to the rapid development of next-generation sequencing, abundant genomic microsatellites with a high level of polymorphisms are available for plant species (Xia et al., 2016). Based on complete plastomes, numerous cpSSRs were detected for the five Chaihu germplasm resources, varying from 51 (*B. chinense* BC05) to 81 (*B. marginatum* var. *stenophyllum*). The mononucleotide category was the most abundant, followed by di-, tri-, tetra- and pentanucleotides. Further, seven cpSSRs were found to be polymorphic across the five *Bupleurum* species, and were located in noncoding regions. One polymorphic cpSSR was located in *ndh* C-*trn* V/UAC, which also exhibited high nucleotide diversity. We propose that *ndh* C-*trn* V/UAC might be candidate molecular markers for cultivated *Bupleurum* species. At the same time, a total of 438 candidate polymorphic nSSRs were detected, most of which were dimers. Among polymorphic cpSSRs and nSSRs, the di- and trinucleotide repeats were dominant categories and no monomers were detected, consistent with the characteristics of the identified polymorphic SSRs in other taxonomic studies (Bhandari et al., 2020; Dabral et al., 2021). Notably, all regions with high variability possessed SSRs. The identified SSRs are helpful in intraspecific phylogeographic and population-level genetic studies of Chaihu germplasms as in *Angelica heterocarpa* (Revardel & Lepais, 2022), *Grevillea robusta* (Dabral et al., 2021), and *Erysimum teretifolium* (del Valle, Herman & Whittall, 2020).

### Adaptive evolution among *Bupleurum* species

Plants might leave fingerprints in plastid genomes in response to environmental changes, within which some genes exhibit positive selection. The value of *Ka*/*Ks* is used to measure the selective pressure of the coding genes in angiosperms (Yang & Nielsen, 2002). The chloroplast functions directly in photosynthesis and carbon fixation, and three genes with essential roles in photosynthesis showed positive selection in this study. Among five *Bupleurum* species, the *Ka*/*Ks* values of *psa* J and *ndh* B were greater than one. Among the plastomes of *B. chinense*, we identified *acc* D as a positively selected gene. The substitution rates of the functional genes at intraspecies and interspecies levels were different, which might be caused by the diverse evolutionary history of *Bupleurum* species. Interestingly, the nucleotide diversity of *psa* J, *ndh* B, and *acc* D in coding regions was not significant (Figs. 3 and 4), presumably due to the close affinity among the *Bupleurum* species. Similar results were found in Huang et al. (2021) and Li et al. (2020). The *psa* J gene is involved in the excitation of photosystem I, which has been essential during the life history of plants (Schöttler et al., 2007). The *ndh* B gene encodes for the subunits of NAD(P)H dehydrogenase complex involved in photosystem I cyclic and chlororespiratory electron transport in higher plants (Martín & Sabater, 2010). Horváth et al. (2000) showed that *ndh* B-inactivated tobacco plants cause a moderate decline in photosynthesis *via* stomatal closure under humidity stress conditions. The *acc* D gene, encoding the *β*-carboxyl transferase subunit of acetyl-CoA-carboxylase, has been demonstrated as an essential gene in plastid genome evolution (Kode et al., 2005). Madoka et al. (2002) confirmed that *acc* D affected leaf longevity and seed yield.

*B. chinense* is a widespread species in China, within which three forms were recognized by Sheh & Watson (2005) with differences in leaves and bracteoles. Environmental variation drives leaf morphology variation within species (Byars, Papst & Hoffmann, 2007). We also observed leaf variations in the *B. chinense* populations under different ecological conditions during field sampling. Han et al. (2006) identified three types of *B. chinense* based on leaf shapes, namely broad, long, and medium. The mutation accumulation of functional genes affects photosynthesis efficiency, which may cause ecological diversity of plants (Zheng et al., 2017). Consequently, positively selected genes provide important insights into adaptive molecular evolution of *Bupleurum* species.

## Conclusions

In this study, the complete chloroplast genomes and nuclear ITS of five Bupleuri Radix germplasm resources, namely *B. bicaule*, *B. chinense*, *B. falcatum*, *B. marginatum* var. *stenophyllum*, and *B. scorzonerifolium*, were obtained using genome skimming. All plastomes we sequenced were conserved and exhibited a typical quadripartite structure, encoding 113 identical genes including 79 protein-coding genes, 30 tRNA genes, and four rRNA genes. Phylogenetic analysis inferred using the chloroplast genomes and nrITS resolved the relationships of the five *Bupleurum* species and their closely related species. *B. marginatum* var. *stenophyllum* was separate from the other four *Bupleurum* species studied. *B. chinense* and *B. scorzonerifolium*, which are the official botanical origins of Chaihu in the Chinese Pharmacopoeia, were in two separate clades. However, the phylogenetic delimitation of *B. bicaule* and *B. falcatum* needs further study, because introgressive hybridization might be involved. By comparison analysis, molecular markers, including plastid hotspots and polymorphic SSRs, were generated for authentication of Chaihu germplasms. Photosynthesis related genes *psa* J, *ndh* B, and *acc* D were found to be under positive selection as a response to adapting to diverse environments.

##  Supplemental Information

10.7717/peerj.15157/supp-1 Supplemental Information 1 The information of 8 Bupleurum individuals sequenced in the study

10.7717/peerj.15157/supp-2 Supplemental Information 2 List of genes encoded in the plastomes in the study

10.7717/peerj.15157/supp-3 Supplemental Information 3 The numbers of SNPs and InDels across Bupleurum plastomes with BC01 as a reference

10.7717/peerj.15157/supp-4 Supplemental Information 4 CpSSRs of five Bupleurum species

10.7717/peerj.15157/supp-5 Supplemental Information 5 Polymorphic cpSSRs of five Bupleurum species

10.7717/peerj.15157/supp-6 Supplemental Information 6 Polymorphic nSSRs of five Bupleurum species

10.7717/peerj.15157/supp-7 Supplemental Information 7 The complete chloroplast genome of *B. chinense* BC02

10.7717/peerj.15157/supp-8 Supplemental Information 8 The complete chloroplast genome of *B. chinense* BC01

10.7717/peerj.15157/supp-9 Supplemental Information 9 The complete chloroplast genome of *B. chinense* BC03

10.7717/peerj.15157/supp-10 Supplemental Information 10 The complete chloroplast of *B. chinense* BC04

10.7717/peerj.15157/supp-11 Supplemental Information 11 The complete chloroplast genome of *B. chinense* BC05

10.7717/peerj.15157/supp-12 Supplemental Information 12 The complete chloroplast genome of *B. scorzonerifolium* BS01

10.7717/peerj.15157/supp-13 Supplemental Information 13 The complete chloroplast genome of *B. scorzonerifolium* BS02

10.7717/peerj.15157/supp-14 Supplemental Information 14 The complete chloroplast genome of *B. bicaule* BB01

## Additional Information and Declarations

### Field Study Permissions

The following information was supplied relating to field study approvals (i.e., approving body and any reference numbers): Field experiments were approved by the Research Council of the Xianyang Normal University (project number: 2019012).

### DNA Deposition

The following information was supplied regarding the deposition of DNA sequences: The chloroplast genomes are available at NCBI: *B. chinense* genomes under NCBI NO. ON553226–ON553230, *B. scorzonerifolium* genomes under NCBI NO. ON553232–ON553233, *B. bicaule* under NCBI NO. ON553231.

### Data Availability

The following information was supplied regarding data availability: The raw data are available in the Supplemental Files.

## References

[ref-1] Amiryousefi A, Hyvönen J, Poczai P (2018). IRscope: an online program to visualize the junction sites of chloroplast genomes. Bioinformatics.

[ref-2] Baldwin BG, Sanderson MJ, Porter JM, Wojciechowski MF, Campbell CS, Donoghue MJ (1995). The ITS region of nuclear ribosomal DNA: a valuable source of evidence on angiosperm phylogeny. Annals of the Missouri Botany Garden.

[ref-3] Bankevich A, Nurk S, Antipov D, Gurevich AA, Dvorkin M, Kulikov AS, Lesin VM, Nikolenko SI, Pham S, Prjibelski AD, Pyshkin AV, Sirotkin AV, Vyahhi N, Tesler G, Alekseyev MA, Pevzner PA (2012). SPAdes: a new genome assembly algorithm and its applications to singlecell sequencing. Journal of Computational Biology.

[ref-4] Bhandari MS, Meena RK, Shamoon A, Saroj S, Kant R, Pandey S (2020). First de Novo genome specific development, characterization and validation of simple sequence repeat (SSR) markers in genus *Salvadora*. Molecular Biology Reports.

[ref-5] Borsch T, Quandt D (2009). Mutational dynamics and phylogenetic utility of noncoding chloroplast DNA. Plant Systematics and Evolution.

[ref-6] Byars SG, Papst W, Hoffmann AA (2007). Local adaptation and cogradient selection in the alpine plant, Poa hiemata, along a narrow altitudinal gradient. Evolution.

[ref-7] Chao Z, Zeng W, Liao J, Liu L, Liang Z, Li X (2014). DNA barcoding Chinese medicinal *Bupleurum*. Phytomedicine.

[ref-8] Dabral A, Shamoon A, Meena RK, Kant R, Pandey S, Ginwal HS, Bhandari MS (2021). Genome skimming-based simple sequence repeat (SSR) marker discovery and characterization in *Grevillea Robusta*. Physiology and Molecular Biology of Plants.

[ref-9] Davis CC, Xi Z, Mathews S (2014). Plastid phylogenomics and green plant phylogeny: almost full circle but not quite there. BMC Biology.

[ref-10] Del Valle JC, Herman JA, Whittall JB (2020). Genome skimming and microsatellite analysis reveal contrasting patterns of genetic diversity in a rare sandhill endemic (*Erysimum Teretifolium*, Brassicaceae). PLOS ONE.

[ref-11] Delsuc F, Brinkmann H, Philippe H (2005). Phylogenomics and the reconstruction of the tree of life. Natural Reviews Genetics.

[ref-12] Dodsworth S (2015). Genome skimming for next-generation biodiversity analysis. Trends in Plant Science.

[ref-13] Dong W, Xu C, Li C, Sun J, Zuo Y, Shi S, Cheng T, Guo J, Zhou S (2015). Ycf1, the most promising plastid DNA barcode of land plants. Scientific Reports.

[ref-14] Frazer KA, Pachter L, Poliakov A, Rubin EM, Dubchak I (2004). VISTA: computational tools for comparative genomics. Nucleic Acids Research.

[ref-15] Fu CN, Mo ZQ, Yang JB, Cai J, Ye LJ, Zou JY, Qin HB, Zheng W, Hollingsworth PM, Li DZ, Gao LM (2022). Testing genome skimming for species discrimination in the large and taxonomically difficult genus *Rhododendron*. Molecular Ecology Resources.

[ref-16] George B, Bhatt BS, Awasthi M, George B, Singh AK (2015). Comparative analysis of microsatellites in chloroplast genomes of lower and higher plants. Current Genetics.

[ref-17] Han M, Wang SJ, Yang LM, Wang XQ, Zhang LX (2006). Contrastive study on main genetic characters of aberrance types of *Bupleurum chinense*. Journal of Jilin Agricultural University.

[ref-18] Horváth EM, Peter SO, Joët T, Rumeau D, Cournac L, Horváth GV, Kavanagh TA, Schäfer C, Peltier G, Medgyesy P (2000). Targeted inactivation of the plastid *ndhB* gene in Tobacco results in an enhanced sensitivity of photosynthesis to moderate stomatal closure. Plant Physiology.

[ref-19] Huang R, Xie X, Chen A, Li F, Tian E, Chao Z (2021). The chloroplast genomes of four *Bupleurum* (Apiaceae) species endemic to Southwestern China, a diversity center of the genus, as well as their evolutionary implications and phylogenetic inferences. BMC Genomics.

[ref-20] Huang W, Sun R (2010). Study on hepatotoxicity on rats caused by crude extracts of total saikosaponins and correlation with oxidative damage mechanism. Zhongguo Zhong Yao Za Zhi.

[ref-21] Katoh K, Misawa K, Kuma K, Miyata T (2002). MAFFT: a novel method for rapid multiple sequence alignment based on fast fourier transform. Nucleic Acids Research.

[ref-22] Kearse M, Moir R, Wilson A, Stones-Havas S, Cheung M, Sturrock S, Buxton S, Cooper A, Markowitz S, Duran C (2012). Geneious Basic: an integrated and extendable desktop software platform for the organization and analysis of sequence data. Bioinformatics.

[ref-23] Kode V, Mudd EA, Iamtham S, Day A (2005). The tobacco plastid *acc* D gene is essential and is required for leaf development. Plant Journal.

[ref-24] Krzywinski M, Schein J, Birol I, Connors J, Gascoyne R, Horsman D, Jones SJ, Marra MA (2009). Circos: an information aesthetic for comparative genomics. Genome Research.

[ref-25] Kumar S, Stecher G, Li M, Knyaz C, Tamura K (2018). MEGA X: molecular evolutionary genetics analysis across computing platforms. Molecular Biology and Evolution.

[ref-26] Lahaye R, Savolainen V, Duthoit S, Maurin O (2008). A test of *psb* K-*psb* I and *atp* F-*atp* H as potential plant DNA barcodes using the flora of the Kruger National Park as a model system (South Africa). Nature Precedings.

[ref-27] Langmead B, Salzberg SL (2012). Fast gapped-read alignment with Bowtie 2. Nature Methods.

[ref-28] Li J, Xie DF, Guo XL, Zheng ZY, He XJ, Zhou SD (2020). Comparative analysis of the complete plastid genome of five *Bupleurum* species and new insights into DNA barcoding and phylogenetic relationship. Plants.

[ref-29] Librado P, Rozas J (2009). DnaSP v5: a software for comprehensive analysis of DNA polymorphism data. Bioinformatics.

[ref-30] Lin TP, Chuang WJ, Huang SS, Hwang SY (2003). Evidence for the existence of some dissociation in an otherwise strong linkage disequilibrium between mitochondrial and chloroplastic genomes in *Cyclobalanopsis glauca*. Molecular Ecology.

[ref-31] Liu B, Ma Z, Ren C, Hodel RGJ, Sun M, Liu X, Liu G, Hong D, Zimmer EA, Wen J (2021a). Capturing single-copy nuclear genes, organellar genomes, and nuclear ribosomal DNA from deep genome skimming data for plant phylogenetics: a case study in Vitaceae. Journal of Systematics and Evolution.

[ref-32] Liu LX, Du YX, Folk RA, Wang SY, Soltis DE, Shang FD, Li P (2020). Plastome evolution in Saxifragaceae and multiple plastid capture events involving *Heuchera* and *Tiarella*. Frontiers in Plant Science.

[ref-33] Liu W, Cheng X, Kang R, Wang Y, Guo X, Jing W, Wei F, Ma S (2021b). Systematic characterization and identification of Saikosaponins in extracts from *Bupleurum marginatum* Var, stenophyllum using UPLC-PDA-Q/TOF-MS. Frontiers in Chemistry.

[ref-34] Luo RB, Liu BH, Xie YL, Li ZY, Huang WH, Yuan JY, He GZ, Chen YX, Pan Q, Liu YJ, Tang JB, Wu GX, Zhang H, Shi YJ, Liu Y, Yu C, Wang B, Lu Y, Han CL, Cheung DW, Yiu SM, Peng SL, Zhu XQ, Liu GM, Liao XK, Li YR, Yang HM, Wang J, Lam TW, Wang J (2012). SOAPdenovo2: an empirically improved memory-efficient short-read de novo assembler. GigaScience.

[ref-35] Lv L, Huang W, Yu X, Ren H, Sun R (2009). Comparative research of different *Bupleurum chinense* composition to influence of hepatotoxicity of rats and oxidative damage mechanism. Zhongguo Zhong Yao Za Zhi.

[ref-36] Ma CD, Yang YC, Chang H, Wang EH, Hui SL, Sun Y, Wang CL, Liu F (2020). Textual research and thinking of industry development on Shaanxi Buleuri Radix. Modern Chinese Medicine.

[ref-37] Ma XG, Zhao C, Wang CB, Liang QL, He XJ (2015). Phylogenetic analyses and chromosome counts reveal multiple cryptic species in *Bupleurum Commelynoideum* (Apiaceae): cryptic species in *Bupleurum Commelynoideum*. Journal of Systematics and Evolution.

[ref-38] Madoka Y, Tomizawa KI, Mizoi J, Nishida I, Nagano Y, Sasaki Y (2002). Chloroplast transformation with modified *acc* D operon increases acetyl-CoA carboxylase and causes extension of leaf longevity and increase in seed yield in tobacco. Plant Cell Physiology.

[ref-39] Martín M, Sabater B (2010). Plastid *ndh* genes in plant evolution. Plant Physiology and Biochemistry.

[ref-40] Meng KK, Chen SF, Xu KW, Zhou RC, Li MW, Dhamala MK, Liao WB, Fan Q (2021). Phylogenomic analyses based on genome-skimming data reveal cyto-nuclear discordance in the evolutionary history of *Cotoneaster* (Rosaceae). Molecular Phylogenetics and Evolution.

[ref-41] National Pharmacopoeia Committee (2020). Pharmacopoeia of the People’s Republic of China.

[ref-42] Neves SS, Watson MK (2004). Phylogenetic relationships in *Bupleurum* (Apiaceae) based on nuclear ribosomal DNA ITS sequence data. Annals of Botany.

[ref-43] Palmer JD (1985). Comparative organization of chloroplast genomes. Annual Review of Genetics.

[ref-44] Pan SL, Shun Q, Bai QM, Bao XS (2002). The coloured atlas of the medicinal plants from genus Bupleurum in China.

[ref-45] Parks M, Cronn R, Liston A (2009). Increasing phylogenetic resolution at low taxonomic levels using massively parallel sequencing of chloroplast genomes. BMC Biology.

[ref-46] Perry AS, Wolfe KH (2002). Nucleotide substitution rates in Legume chloroplast DNA depend on the presence of the inverted repeat. Journal of Molecular Evolution.

[ref-47] Posada D (2008). jModelTest: phylogenetic model averaging. Molecular Biology and Evolution.

[ref-48] Rambaut A (2018). http://tree.bio.ed.ac.uk/software/figtree.

[ref-49] Revardel E, Lepais O (2022). Development and characterization of microsatellite markers for the French endemic *Angelica Heterocarpa* (Apiaceae) and congeneric sympatric species. BMC Research Notes.

[ref-50] Ronquist F, Huelsenbeck JP (2003). MrBayes 3: Bayesian phylogenetic inference under mixed models. Bioinformatics.

[ref-51] Schöttler MA, Flügel C, Thiele W, Stegemann S, Bock R (2007). The plastome-encoded *psa* J subunit is required for efficient photosystem I excitation, but not for plastocyanin oxidation in Tobacco. Biochemical Journal.

[ref-52] Shaw J, Lickey EB, Schilling EE, Small RL (2007). Comparison of whole chloroplast genome sequences to choose noncoding regions for phylogenetic studies in Angiosperms: the Tortoise and the Hare III. American Journal of Botany.

[ref-53] Sheh ML, Watson MF, Wu ZY, Raven PH (2005). Bupleurum. Flora of China.

[ref-54] Stamatakis A (2014). RAxML version 8: a tool for phylogenetic analysis and post-analysis of large phylogenies. Bioinformatics.

[ref-55] Straub SC, Parks M, Weitemier K, Fishbein M, Cronn RC, Liston A (2012). Navigating the tip of the genomic iceberg: next-generation sequencing for plant systematics. American Journal of Botany.

[ref-56] Tang JB, Xia HA, Cao ML, Zhang XQ, Zeng WY, Hu SN, Tong W, Wang J, Wang J, Yu J, Yang HM, Zhu LH (2004). A comparision of rice chloroplast genomes. Plant Physiology.

[ref-57] Thakur VV, Tiwari S, Tripathi N, Tiwari G (2019). Molecular identification of medicinal plants with amplicon length polymorphism using universal DNA barcodes of the *atp* F-*atp* H, trnL and *trn* H-*psb* A regions. 3 Biotech.

[ref-58] The Society of Japanese Pharmacopoeia (2011). The Japanese Pharmacopoeia.

[ref-59] Thiel T, Michalek W, Varshney RK, Graner A (2003). Exploiting EST databases for the development and characterization of gene derived SSR-markers in barley (*Hordeum vulgare* L.). Theoretical and Applied Genetics.

[ref-60] Thode VA, Lohmann LG, Sanmartín I (2020). Evaluating character partitioning and molecular models in plastid phylogenomics at low taxonomic levels: a case study using *Amphilophium* (Bignonieae, Bignoniaceae). Journal of Systematics and Evolution.

[ref-61] Wakasugi T, Tsudzuki J, Ito S, Nakashima K, Tsudzuki T, Sugiura M (1994). Loss of all *ndh* genes as determined by sequencing the entire chloroplast genome of the black pine *Pinus thunbergii*. Proceedings of the National Academy of Sciences of the United States of America.

[ref-62] Wang CB, Ma XG, He XJ (2011). A taxonomic re-Assessment in the Chinese *Bupleurum* (Apiaceae): insights from morphology, nuclear ribosomal internal transcribed spacer, and chloroplast (*trn* H-*psb* A, matK) sequences. Journal of Systematics and Evolution.

[ref-63] Wang H, Feng ML, Zhang Y, Xi XH, Zhang XH (2020). Comparative study on acute toxicity, antipyretic and anti-inflammatory effects of *Bupleurum marginatum* var. stenophyllum and *B. chinense*. World Science and Technology- Modernization of Traditional Chinese Medicine and Materia Medica.

[ref-64] Wang QZ, Zhou SD, Liu TY, Pang YL, Wu YK, He XJ (2008). Phylogeny and classification of Chinese Bupleurum based on nuclear ribosomal DNA internal transcribed spacer and rps16. Journal of Systematics and Evolution.

[ref-65] Wendel JF, Doyle JJ, Soltis DE, Soltis PS, Doyle JJ (1998). Phylogenetic incongruence: windows into genome history and molecular evolution. Molecular systematics of plants II DNA sequencing.

[ref-66] Weng ML, Blazier JC, Govindu M, Jansen RK (2014). Reconstruction of the ancestral plastid genome in Geraniaceae reveals a correlation between genome rearrangements, repeats, and nucleotide substitution rates. Molecular Biology and Evolution.

[ref-67] Wikström N, Bremer B, Rydin C (2020). Conflicting phylogenetic signals in genomic data of the coffee family (Rubiaceae). Journal of Systematics and Evolution.

[ref-68] Wolfe KH, Morden CW, Palmer JD (1992). Function and evolution of a minimal plastid genome from a nonphotosynthetic parasitic plant. Proceedings of the National Academy of Sciences of the United States of America.

[ref-69] Wu CS, Wang YN, Liu SM, Chaw SM (2011). Comparative chloroplast genomes of Pinaceae: insight into the mechanism of diversified genomic organizations. Genome Biology and Evolution.

[ref-70] Xia EH, Yao QY, Zhang HB, Jiang JJ, Zhang LP, Gao LZ (2016). CandiSSR: an efficient pipeline used for identifying candidate polymorphic SSRs based on multiple assembled sequences. Frontiers in Plant Science.

[ref-71] Xia ZD, Liu X, Tong LG, Wang H, Feng ML, Xi XH, He P, Qin XM (2021). Comparison of chemical constituents of *Bupleurum marginatum* var. stenophyllum and *B. chinense* DC. using UHPLC-Q-TQF-MS based on a metabonomics approach. Biomedical Chromatography.

[ref-72] Xie H, Huo K, Chao Z, Pan S (2009). Identification of crude drugs from Chinese medicinal plants of the genus *Bupleurum* using ribosomal DNA ITS sequences. Planta Medica.

[ref-73] Xie XN, Huang R, Li F, Tian EW, Li C, Chao Z (2021). Phylogenetic position of *Bupleurum sikangense* inferred from the complete chloroplast genome sequence. Gene.

[ref-74] Xu N, Shi YN, Zhong X, Cao Y, Wang L, Jia TZ (2014). A new saikogenin from the roots of *Bupleurum bicaule*. Chinese Journal of Natural Medicines.

[ref-75] Yang F, Dong X, Yin X, Wang W, You L, Ni J (2017). Radix Bupleuri a review of traditional uses, botany, phytochemistry, pharmacology, and toxicology. Biomed Research International.

[ref-76] Yang ZH, Nielsen R (2002). Codon-substitution models for detecting molecular adaptation at individual sites along specific lineages. Molecular Biology and Evolution.

[ref-77] Yao H, Song J, Liu C, Luo K, Han J, Li Y, Pang XH, Xu HX, Zhu YJ, Xiao PG, Chen SL (2010). Use of ITS2 region as the universal DNA barcode for plants and animals. PLOS ONE.

[ref-78] Zhang GX, Wang H, Liu Y, Yao RY, Jiang JM, Wang QL, Wei JH (2021). Survey and analysis of cultivated *Bupleurum* spp., germplasm resources in China. Modern Chinese Medicine.

[ref-79] Zhang GX, Wang H, Shi LC, Liu Y, Yao RY, Sui C, Yang CM, Ji HL, Wang QL, Wei JH (2022). Identification of the original plants of cultivated Bupleuri Radix based on DNA barcoding and chloroplast genome analysis. PeerJ.

[ref-80] Zheng XM, Wang JR, Feng L, Liu S, Pang HB, Qi L, Li J, Sun Y, Qiao WH, Zhang LF, Cheng YL, Yang QW (2017). Inferring the evolutionary mechanism of the chloroplast genome size by comparing whole-chloroplast genome sequences in seed plants. Scientific Reports.

[ref-81] Zhou P, Shi W, He XY, Du QY, Wang F, Guo J (2021). Saikosaponin D: review on the antitumour effects, toxicity and pharmacokinetics. Pharmaceutical Biology.

[ref-82] Zhu L, Liang ZT, Yi T, Ma Y, Zhao ZZ, Guo BL, Zhang JY, Chen HB (2017). Comparison of chemical profiles between the root and aerial parts from three *Bupleurum* species based on a UHPLC-QTOF-MS metabolomics approach. BMC Complementary and Alternative Medicine.

